# Time-trends in the utilization of decentralized mental health services in Norway - A natural experiment: The VELO-project

**DOI:** 10.1186/1752-4458-4-5

**Published:** 2010-03-31

**Authors:** Lars H Myklebust, Knut W Sørgaard, Svein Bjorbekkmo, Martin R Eisemann, Reidun Olstad

**Affiliations:** 1 Psychiatric Research Centre of Northern Norway, University Hospital of North Norway, Tromsø, Norway; 2 The Nordland Hospital Trust, Bodø, Norway; 3 The University of Tromsø, Institute of Psychology, Faculty of Health Sciences, Tromsø, Norway; 4 The University of Tromsø, Institute of Clinical Medicine, Faculty of Health Sciences, Tromsø, Norway

## Abstract

**Background:**

There are few reports on the effects of extensive decentralization of mental health services. We investigated the total patterns of utilization in a local-bed model and a central-bed model.

**Methods:**

In a time-trend case-registry design, 7635 single treatment episodes, from the specialist and municipality services in 2003-2006, were linked to 2975 individual patients over all administrative levels. Patterns of utilization were analyzed by univariate comparisons and multivariate regressions.

**Results:**

Total treated prevalence was consistently higher for the central-bed system. Outpatient utilization increased markedly, in the central-bed system. Utilization of psychiatric beds decreased, only in the central-bed system. Utilization of highly supported municipality units increased in both systems. Total utilization of all types of services, showed an additive pattern in the local-bed system and a substitutional pattern in the central-bed system. Only severe diagnoses predicted inpatient admission in the central-bed system, whereas also anxiety-disorders and outpatient consultations predicted inpatient admission in the local-bed system. Characteristics of the inpatient populations changed markedly over time, in the local-bed system.

**Conclusions:**

Geographical availability is not important as a filter in patients' pathway to inpatient care, and the association between distance to hospital and utilization of psychiatric beds may be an historical artefact. Under a public health-insurance system, local psychiatric personnel as gatekeepers for inpatient care may be of greater importance than the availability of local psychiatric beds. Specialist psychiatric beds and highly supported municipality units for people with mental health problems do not work together in terms of utilization. Outpatient and day-hospital services may be filters in the pathway to inpatient care, however this depends on the structure of the whole service-system. Local integration of psychiatric services may bring about additive, rather than substitutional patterns of total utilization. A large proportion of decentralized psychiatric beds may hinder the development of various local psychiatric services, with negative consequences for overall treated prevalence.

## Introduction

The deinstitutionalization of western mental health care since the 1950's, has been associated with an increased quality of life and satisfaction with new community services for many patients [1]. It can also be attributed to increased mortality and "transhospitalisations" of former inpatients to marginally staffed services, jail and homelessness [2-4]. Contemporary research, therefore, advocates a balanced approach that includes both mental hospitals and outpatient local community services [5].

The decentralized Norwegian mental health services represent an alternative to the central mental hospitals, were inpatient care are mainly to take place locally, in a system of small units at community mental health centres [6,7]. Although this type of organization may be advantageous, it has largely been left scientifically unnoticed. In an extensive literature search, only one relevant article was found [8]. Further, none were found on the dynamics between local psychiatric beds and other local mental health services. The decentralized Norwegian Mental Health Services, therefore, represent a relevant scientific focus.

### Norwegian Mental Health Services

After a slow and uncoordinated initial phase of deinstitutionalisation [9], a white paper on a national public system of mental health services was presented to The Norwegian Parliament [10]. The system were divided in three administrative levels:

• The 1^st ^level of Municipality services, staffed by semi-specialized personnel and GPs.

• The 2^nd ^level of local Community Mental Health Centres (CMHC).

• The 3^rd ^level of Central Mental Hospitals (CMH). Both the 2^nd ^and 3^rd ^level-services are staffed with psychiatrists, clinical psychologists and specialist nurses.

The CMHC are the core component of the system, with duties to provide and coordinate services in geographically defined sectors [7]. Local adaptation has led to diversity regarding their structure and size, keeping, within the frames of national policies of health insurance, case registration, and clinical standards [11].

### The VELO-project

In 2005, the neighbouring CMHCs of Vesterålen and of Lofoten drew scientific attention because of noticeable organizational dissimilarities, whilst at the same time a strong resemblance in the catchment areas' characteristics and needs [12]. This opportunity to study mental health services, in a close to natural experiment, has been well described in a previous publication [13].

In short, the main differences between the two CMHCs are in their number of outpatient services and local psychiatric beds. The CMHC of Vesterålen has 1 outpatient clinic (which incorporates both -consultations and various forms of day-care treatments), and 20 psychiatric beds at 3 fully staffed units. The CMHC of Lofoten has 2 outpatient clinics (1 until 2005) and 2 fully staffed day-hospitals (1 until 2005), and a maximum of 6 beds available at the local general hospital. Consequently, the staff sizes of the centres are respectively 77 versus 29 man-labour-years. Both may also refer patients to the Nordland County's only Central Mental Hospital (CMH) located in the distant town of Bodø.

**Figure 1:**
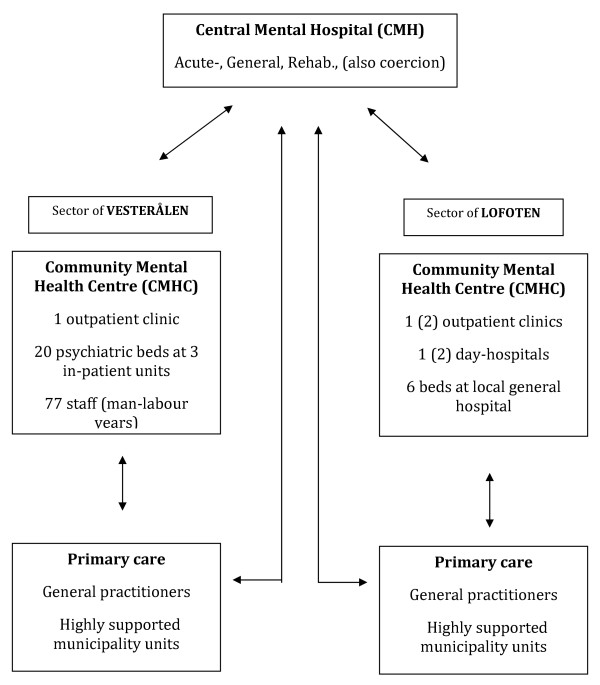
**Outline of the total mental health services in the two sectors of Vesterålen and Lofoten, County of Nordland, Norway**.

In the following, the service-system of Vesterålen will be termed as a "local-bed model", in contrast to a "central-bed model" of Lofoten. Their outline is illustrated in Figure 1.

### Theoretical perspectives and hypotheses

Several theoretical perspectives may be relevant in the study of mental health services.

The influential *Stage model *on patients' pathways to care, is built upon 5 sequential stages, with 4 corresponding filters of selective permeability [14]. Of particular interest for our study is the 4^th^-filter between specialist outpatient services and hospitals, which regulates the flow of patients in and out of psychiatric beds. One determinant of its permeability, is the availability of services, were the inverse relationship of geographical distance and bed-utilization is known as " Jarvis' Law" [15,16]. Another is the work of outpatient services, although the literature is inconclusive as to whether these reduce or increase bed utilization [17,18].

The recent *Hydraulic model *[19] postulates that the "pressure of morbidity" will tend to equalize throughout a system of services and that the behaviours of one component will affect (all) the others. There are, to our knowledge, no studies that have explicitly tested for the implications of this model. With regard to our study, the model may broaden the dynamic scope into also consider the highly supported municipality units' relation with specialist psychiatric services.

A somewhat different line of reasoning stems from the phenomenon of "supply-induced demand", that suggests a positive association between provision and utilization of hospital beds [20,21]. This phenomenon is also suggested to be relevant for mental health inpatient care [22], but how it is affected by activities of complementary services has not been studied.

### Aims

The aim of this study was to investigate the utilization of different types of mental health services, and the association between them. We formulated the following scientific questions, and deduced specific hypotheses from the relevant theories:

1. Is utilization of psychiatric beds affected by geographic distance to services?

a. *Hypothesis: *Utilization of psychiatric beds will be continually higher in the local-bed system than in the central-bed system.

2. Is utilization of psychiatric beds affected by the development of highly supported municipality units?

a. *Hypothesis: *Increased activities of highly supported municipality units will be associated with a decrease in utilization of psychiatric beds in both systems.

3. Is utilization of psychiatric beds affected by changes in the activities of specialist outpatient- and day-hospital services?

a. *Hypothesis: *An increase in utilization of outpatient services and day-hospitals will be associated with a decrease in utilization of psychiatric beds in both systems.

b. *Hypothesis: *A change in utilization of outpatient services will be associated with altered indications for inpatient admission in both systems.

c. *Hypothesis: *Total utilization of services will be higher for patients in a local-bed system than a central-bed system.

## Methods

The study had a time-trend case-registry design, with an observational period from 2003-2006. Approvals were given from The Regional Ethics Committee for Research and the Norwegian Data Protection Agency. The Directorate for Health and Social Affairs released all health professionals from their confidentiality obligations.

### The psychiatric services

A standard sample of socio-demographic variables, clinical information, type and amount of treatment, is routinely recorded for all patients referred to the Norwegian specialist psychiatric services. These registries were linked across service-levels by use of the patients' Social Id-number. This allowed for aggregation of 7635 single treatment episodes for 2979 individual patients. All episodes were calculated into total treatments for each year of the observation period. Missing data was collected from clinical journals.

The sample consisted of patients between the ages of 18 and 65, with a population size of N = 18 265 for the local-bed system and N = 12 733 for the central-bed system. The net change of population size over the observational period was respectively -146 and 0 persons. Patients who belonged to municipalities outside the catchment areas (N = 22) or moved between the two areas (N = 8) were excluded.

The diagnoses were grouped into 4 broad categories according to the ICD-10 manual, except for 9 patients with serious affective disorders who were classified into the category of *psychosis*, due to relevant features of their disorder. In order to obtain an adequate size of categories for analysis, we collapsed less-frequent diagnoses into "others". We retained the category of "psychiatric observation" for patients who had been discharged without a diagnostic conclusion.

#### Municipality services

Data on residents in highly supported municipality units was obtained by questionnaires to the local administrative authorities. The total number of all 27 residents were subsequently linked to the psychiatric case-registries by their social Id-number.

#### Analysis

The dependent variable for analysis was utilization of services. This was defined by the number of patients treated and sum of treatments, for all modalities: inpatient days & nights, outpatient consultations, days in day-hospitals (i.e. various day-care treatments in the local-bed system), and days & nights in highly supported municipality units. In addition, we used the concept of "bed-equivalents" to convey inpatient utilization into terms of single beds. 1 bed-equivalent equals 365 inpatient days & nights, continuous availability assumed.

#### Statistics

The two systems were compared by rates per 1000 inhabitants, for all parameters. Differences were tested by standard procedure for confidence intervals of proportions. No adjustment in population-structures of gender and age was made, due to small and non-significant differences between the two catchment areas [23]. We also performed a univariate analysis of the two systems inpatient-populations at the start (2003) and end (2006) of the observational period. The relation between utilization of psychiatric beds and other types of services was analysed by a series of multivariate logistic regressions, performed for each service-system separately, at the beginning and end of the observational period (2003 and 2006). The dependent variable was whether the patient was hospitalized or not. The covariates were gender, age, diagnosis and utilizations of outpatient consultations or day-hospital. Outpatient consultations and days in day-hospital were log-transformed due to skewed distribution. All covariates were entered simultaneously.

The calculation and statistical analyses was carried out by Microsoft Excel 12.1.3 and SPSS 16.1 software.

## Results

Differences in patterns of utilization between the two systems are reported in tables, while changes within the systems are reported in text only (p-levels).

### Total prevalence of patients treated

The total treated prevalence of patients was consistently higher in the central-bed system, except during 2005. The difference between the two systems increased from 13.8% in 2003 to 17.2% in 2006. Within each system, the net increase was of 14% (p < .000) for the central-bed system, and 10% (p < .05) for the local-bed system. See upper part of Additional file 1.

### Psychiatric beds

For the number of psychiatric inpatients per 1000 inhabitants, there was no difference between the two systems in the observational period, except for a 32% higher rate in the central-bed system in 2004. There was no net change within either of the systems. For bed-equivalents, the difference between the two systems increased from none in 2003, to a 15.5% lower rate for the central-bed system in 2006. Within the systems, the net reduction was of 10.1% (p < .000) for the central-bed system, while no change for the local-bed system (see Additional file 1).

### Highly supported municipality units

For utilization of beds in highly supported municipality units, the local-bed system consistently outnumbered the central-bed system, which did not have such until 2006. Within the systems, the net increase in bed-equivalents was about 40% for the local-bed system, from 0.71 per 1000 in 2003, to 1.04 in 2006 (p < .000). For the central-bed system, the net increase was from 0 per 1000 in 2003, to 0.31 per 1000 in 2006 (see Additional file 1).

### Total inpatient utilization

When psychiatric beds and beds in highly supported municipality units were combined, the local-bed system consistently utilized inpatient care at nearly twice the rate of the central-bed system. For the rate of patients, there was no difference over the observational period, except for a 28% higher rate for the central-bed system in 2004. Within the systems there was no significant net change over the observational period. For bed-equivalents, there was a 45% higher rate for the local-bed system than the central-bed system both in 2003 and 2006. Within the systems, there was a net increase of 24% for the local-bed system, 17% for the central-bed system (p < .000). See lower part of Additional file 1.

### Outpatient consultations

For the ratio of outpatients compared to the total sample of patients, the difference between the two systems changed from an 8% higher rate for the local-bed system in 2003, to none in 2006 Within the systems, there was a net increase of 5% for the central-bed system (p < .000), while there was no change for the local-bed system. For outpatients per 1000 inhabitants, the difference between the two systems increased from none in 2003, to a 15% higher rate for the central-bed system in 2006. Within the systems, there was a net increase of 19% (p < .000) for the central-bed system, while no change for the local-bed system. For the number of consultations per 1000 inhabitants, the difference between the systems was a 28.3% higher rate for the central-bed system in 2003, decreasing to a 22.9% higher rate in 2006. Within the systems, there was a net increase of 5.3% for the central-bed system, and of 10% for the local-bed system (p < .000). See upper part of Additional file 2.

### Day-hospital

For the ratio of day-hospital patients compared to the total sample of patients, there was no difference between the two systems during the observational period. For day-hospital patients per 1000 inhabitants, the difference between the two systems increased over the observational period from none in 2003, to a 90% higher rate for the central-bed system in 2006. Within the systems, there was a net increase of 380% (p < .000) for the central-bed system, while there was no change for the local-bed system. For number of day-hospital days per 1000 inhabitants, the difference between the two systems was reduced over the observational period from 95.0% higher rate for the central-bed system in 2003, to 70.0% in 2006. Within the systems, there was a net increase of 87.5% (p < .000) for the local-bed system and 21.0% (p < .000) for the central-bed system (see Additional file 2).

### Characteristics of inpatient populations

The distribution of age, gender and diagnosis of inpatients at the start (2003) and end (2006) of the observational period are presented in Additional file 3.

For inpatients with psychosis or affective disorders, there were no differences between the two systems.

For inpatients with substance abuse, the central-bed system consistently admitted more than the local-bed system, although the local-bed system more than doubled these admissions (p < .000) over the observational period. There was no change within the central-bed system.

For inpatients with anxiety-disorders, there was no difference between the systems at the beginning of the observational period, however the local bed systems ultimately admitted more than the central-bed system. Within the systems, the central-bed system reduced these admissions with more than 50% (p < .000), while there was no change for the local-bed system.

For gender, the central bed system initially admitted more male inpatients than the local-bed system, however there was no difference at the end of the observational period. Initially there was no difference between the systems used for female inpatients, however the local-bed system admitted more females than the central-bed system at the end of the period. Within the systems there were no significant changes for either gender.

### Predictors of inpatient admission

In order to explore if admission patterns changed during the observational period, logistic regressions were performed, using inpatient admission (yes/no) as the dependent variable. The first (2003) and last (2006) year of the observational period was used for the analyses. The series of logistic regressions on the predictors for psychiatric inpatient admissions were all statistically significant and distinguished between patients being hospitalised or not. See Additional file 4 for the local-bed system, and Additional file 5 for the central-bed system.

### Local-bed system

For 2003, the model of the local-bed system was highly significant at a χ^2 ^109.641, df = 9 (p < .000), and explained between15.0% (Cox and Snell R Square) and 23.5% (Nagelkerke R Square) of the variance. For 2006, the corresponding values were χ^2 ^132.528, df = 9 (p < .000), 16.4% (Cox and Snell R Square) and 25.4% (Nagelkerke R Square). The predictors for inpatient treatment were (in 2003): psychosis, outpatient consultations, age, and psychiatric examination, and (in 2006): psychosis, substance abuse, outpatient consultations, and psychiatric examination.

### Central-bed system

For 2003, the model for the central-bed system was highly significant at a χ^2 ^58.412, df = 9 (p < .000), and explained between 10.4% and 16.0% of the variance. For 2006, the corresponding values were χ^2^116.031, df = 9 (p < .000), 17.4% - 29.2%. The predictors for inpatient treatment were (in 2003): psychosis, substance abuse, and affective disorders, and (in 2006): psychosis, substance abuse, and affective disorders, with the addition of outpatient consultations as a negative predictor.

In sum, the characteristics of inpatient population and predictors for inpatient admissions suggest that indications for utilization of psychiatric beds expanded markedly over time in the local bed model only, and ultimately diverged between the two systems.

## Discussion

Several findings emerged from our comparative study on time-trends of utilization in a "central-bed model" versus a "local-bed model" of services. Treated prevalence was consistently higher for the central-bed system of services. Utilization of highly supported municipality units increased in both systems. Outpatient utilization increased markedly in the central-bed system, while there was a modest increase in the local-bed system. Utilization of psychiatric beds decreased only in the central-bed system. The total pattern of utilization was additive in the local-bed system, in contrast to a more substitutional association in the central-bed system. Only severe diagnoses predicted inpatient admission in the central-bed system, whereas also anxiety-disorders and outpatient consultations predicted inpatient admission in the local-bed system. Characteristics of the inpatient populations changed markedly over time, only in the local-bed system.

### Geographic availability and utilization of psychiatric beds

The substantial changes in utilization of psychiatric beds in the central-bed system over the relative short 4-year observational period, suggest that geographical availability is not important as a filter in patients' pathway to inpatient care. It is in accordance with preliminary findings from Italy [24]. It may be that the longstanding phenomenon of "Jarvis' law" are rooted in data that stem from the beginning of the 1900's [25,26] which can probably not be generalized to contemporary societies.

One interpretation may be that economical factors such as modern health insurance-systems are more crucial than geographical distance for utilization of inpatient services. This further leads to a greater relative importance of local psychiatric personnel as gatekeepers for inpatient care, rather than the presence of local beds. Under a public health-insurance system, local psychiatrists or GPs will certify hospitalization regardless of the individual patients' financial or behavioural abilities for transport.

Utilization of psychiatric beds may therefore rather be considered as affected by other factors that change more rapidly over time than geographic availability, for instance the workings of other services.

### The association between psychiatric beds and beds in highly supported municipality units

The decreasing utilization of psychiatric beds in the central-bed system may be associated with an increase of highly supported municipality units. However, in the local-bed system, utilization of both these types of inpatient services increased over the observational period.

The most plausible interpretation is therefore that utilizations of these two types of services do not affect each other, despite their similar features of 24 hours of care. They may serve different needs, or their use is uncoordinated because they belong to different administrative levels. A hydraulic analogy is consequently of limited relevance for this relation, which may be better characterized by the phenomenon of supply-induced demand. This in accordance with the trend of "reinstitutionalization" evident in recent studies, that have combined utilization of several types residential care [27-29]. Utilization of psychiatric beds may therefore be affected by the activities of other psychiatric services, i.e. outpatient and/or day-hospital treatments.

### The association between psychiatric beds and outpatient services

For the central-bed system, the increase in utilization of outpatient services is contingent with a decrease in utilization of psychiatric beds. For the local-bed system there is instead a positive association in these two types of services. Structural aspects of the whole system of services, rather than only the needs of the patients, may therefore be relevant to explain the diverging results on the relevance of outpatient services as a filter on the pathway to inpatient care [17,22,30,31]. One hypothesis may be that if both in- and outpatient services are integrated in terms of local management or clinical relations, patterns of total utilization will be additive rather than substitutional. This is further supported by our results on the differences in predictors for inpatient admission, were an association between outpatient and inpatient treatment is only found in the local-bed system. Also, the tendency to admit patients with anxiety-disorders and those under psychiatric examination are found only in the local-bed system.

Further, a decentralization of psychiatric beds may be so resource-demanding that they hinder the development of other local services. Small local-bed units with 24 - hour staffing need a relatively high number of personnel organized in shifts. These persons are linked to a geographical site, with specialized buildings and facilities for psychiatric patients. This may decrease the flexibility of the health-care system, and reduce the possibility to convert resources into different types of services. Our results on higher total treated prevalence for the central-bed system compared to the local-bed system support this line of reasoning. The set of services in the central-bed system may be more balanced and relevant to the total needs of the local population, compared to the local-bed systems'. Consequently, heavy emphasis on decentralized psychiatric beds may interfere with the national objectives of increased availability through provision of various local services.

### Strengths and limitations

A major advantage of the study was the opportunity to explore patterns of health service utilization in a close to natural experiment. Both the geographical and demographic similarities of the catchment areas, the absence of private service providers, and the overarching national clinical standards and public health insurances help to rule out confounding variables in interpretation of the results.

A related advantage was the permission to use the 11-digit Social Id-number for all patients. This allowed for analysis on the level of individual patients throughout the total service-system and is to our knowledge the first time in Norwegian health-service research.

The results emerge in the cultural context of the Norwegian society, which may limit generalizations to other countries. These limitations are on the other hand inherent in all health-service research and advice us to interpret all internationally published results cautiously. Our results may be of special relevance to other modern societies with similar structures of settlements and geography.

## Conclusions

Geographical availability is not important as a filter in patients' pathway to inpatient care, and the association between distance to hospital and utilization of psychiatric beds may be an historical artefact. Under a public health-insurance system, local psychiatric personnel as gatekeepers for inpatient care may be of greater importance than the availability of local psychiatric beds. Specialist psychiatric beds and highly supported municipality units for people with mental health problems do not work together in terms of utilization. Outpatient and day-hospital services may be filters in the pathway to inpatient care, however this depends on the structure of the whole service-system. Local integration of psychiatric services may bring about additive, rather than substitutional patterns of total utilization. A large proportion of decentralized psychiatric beds may hinder the development of various local psychiatric services, with negative consequences for overall treated prevalence.

## Competing interests

The authors declare that they have no competing interests.

## Authors' contributions

All authors participated in the design of the study. LHM wrote the manuscript. LHM and RO performed the statistical analysis. All authors read and approved the final manuscript.

## Supplementary Material

Additional file 1 **Utilization of residential treatment/care: psychiatric beds and highly supported living units in the period of 2003 - 2006, comparing a psychiatric local-bed system to a central-bed system.** *P < .001 and **P < .05 when systems are compared. Click here for file

Additional file 2 **Total treated prevalence, utilization of outpatient and day-hospital services over the period of 2003 - 2006, a local-bed system versus a central-bed system.** *P < .001 and **P < .05 when models are compared. Click here for file

Additional file 3 **Inpatient-population characteristics in a local-bed system versus a central-bed system.** The years of 2003 and 2006. Click here for file

Additional file 4 **Predictors of inpatient treatment (no/yes) in a local-bed system.** The years of 2003 and 2006. Logistic regression model. Click here for file

Additional file 5 **Predictors of inpatient treatment (no/yes) in a central-bed system.** The years of 2003 and 2006. Logistic regression model. Click here for file
