# Supplementary material for: Time-trends in the utilization of decentralized mental health services in Norway - A natural experiment: The VELO-project
Source: Int J Ment Health Syst. 2010 Mar 31;4:5. doi: 10.1186/1752-4458-4-5 (PMC2861015; doi:10.1186/1752-4458-4-5)
Supplement: Additional file 1 — Utilization of residential treatment/care: psychiatric beds and highly supported living units in the period of 2003 - 2006, comparing a psychiatric local-bed system to a central-bed system. *P < .001 and **P < .05 when systems are compared. [file 1752-4458-4-5-S1.DOC]

**Additional file 1**

Utilization of residential treatment/care: psychiatric beds and highly supported living units in the period of 2003 – 2006, comparing a psychiatric local-bed system to a central-bed system. *P< .001 and **P< .05 when systems are compared.

|  | | **Local-bed model** | | | | **Central-bed model** | | | |
| --- | --- | --- | --- | --- | --- | --- | --- | --- | --- |
|  |  | **2003** | **2004** | **2005** | **2006** | **2003** | **2004** | **2005** | **2006** |
| **All treatment** | *Patients* (N)   - 1/1000 inhab. | 675  36,8* | 681  37,2** | 722  39,6 | 742  40,8** | 532  41,9** | 556  43,5** | 541  42,5 | 607  47,8** |
| **Psychiatric bed-treatment** | *Patients* (N)   - 1/1000 inhab. | 139  7,6 | 129  7,1** | 141  7,7 | 157  8,6 | 115  8,9 | 131  10,3** | 106  8,3 | 102  8,0 |
|  | *Days & nights* (sum)   - 1/1000 inhab. | 6604  360,0 | 6825  373,1** | 6775  366,5** | 6731  369,7** | 4477  352,2 | 4391  343,7** | 4505  353,8** | 4068  320,0** |
|  | *Bed-equivalents*   - 1/1000 inhab. | 18,1  0,99 | 18,7  1,02** | 18,3  1,00** | 18,4  1,01** | 12,3  0,96 | 12,0  0,94** | 12,3  0,97** | 11,2  0,88** |
| **Highly supported municipality units** | *Patients* (N)   - 1/1000 inhab. | 13  0,71 | 14  0,77 | 15  0,82 | 19  1,04** | -  - | -  - | -  - | 4  0,31** |
|  | *Days & nights* (sum)   - 1/1000 inhab. | 4745  258,6 | 5110  279,4 | 5475  300,6 | 6935  380,9** | -  - | -  - | -  - | 1460  114,9** |
|  | *Bed-equivalents*   - 1/1000 inhab. | 13,0  0,71 | 14,0  0,77 | 15,0  0,82 | 19,0  1,04** | -  - | -  - | -  - | 4,00  0,31** |
| **Total** | *Patients* (N)   - 1/1000 inhab. | 146  8,4 | 136  7,8** | 149  8,6 | 168  9,7 | 115  8,9 | 131  10,3** | 106  8,3 | 104  8,3 |
|  | *Days & nights* (sum)   - 1/1000 inhab. | 11110  605,4** | 11935  652,5** | 12250  672,6** | 13666  750,6** | 4716  371,0** | 4391  343,7** | 4505  353,8** | 5528  434,9** |
|  | *Bed-equivalents*   - 1/1000 inhab. | 30,4  1,66** | 32,7  1,79** | 33,6  1,84** | 37,4  2,06** | 12,9  1,02** | 12,0  0,94** | 12,3  0,97** | 15,2  1,19** |
