# Supplementary material for: Time-trends in the utilization of decentralized mental health services in Norway - A natural experiment: The VELO-project
Source: Int J Ment Health Syst. 2010 Mar 31;4:5. doi: 10.1186/1752-4458-4-5 (PMC2861015; doi:10.1186/1752-4458-4-5)
Supplement: Additional file 2 — Total treated prevalence, utilization of outpatient and day-hospital services over the period of 2003 - 2006, a local-bed system versus a central-bed system. *P < .001 and **P < .05 when models are compared. [file 1752-4458-4-5-S2.DOC]

**Additional file 2**

Total treated prevalence, utilization of outpatient and day-hospital services over the period of 2003 – 2006, a local-bed system versus a central-bed system. *P < .001 and **P < .05 when models are compared.

| **Treatment modality ^1^** | | **Local-bed model** | | | | **Central-bed model** | | | |
| --- | --- | --- | --- | --- | --- | --- | --- | --- | --- |
|  |  | **2003** | **2004** | **2005** | **2006** | **2003** | **2004** | **2005** | **2006** |
| **All** | *Patients* (N) – total   - 1/1000 inhab. | 675  36,8* | 681  37,2** | 722  39,6 | 742  40,8** | 532  41,9** | 556  43,5** | 541  42,5 | 607  47,8** |
| **Outpatient services** | *Consultations*  Patients (n)   - % of all patients - 1/1000 inhab.   Consultations   - Mean/patient - 1/1000 inhab. | 637  94,4**  34,7  3472  5,5**  189,2** | 639  93,8**  34,9  4333  6,8  236,9 | 683  94,6**  37,5  4227  6,2  232,1 | 685  92,3  37,6**  3789  5,5  208,1** | 462  86,8 **  36,4  3086  6,7*  242,8* | 477  85,8 **  37,3  2903  6,1  227,2 | 478  88,4 **  37,5  2884  6,0  226,5 | 554  91,3    43,6**  3250  5,9  255,7** |
|  | *Day hospital*  Patients (n)   - % of all patients - 1/1000 inhab.   Days   - Mean/patient - 1/1000 inhab. | 17  2,5  0,9  36  2,1**  1,9** | 41  6,0  2,2  218  5,3**  11,9** | 27  3,7  1,5  206  7,6**  11,3** | 27  3,6  1,5**  289  10,7  15,9** | 9  1,7  0,7  550  61,1**  43,3** | 24  4,3  1,9  714  29,8**  55,9** | 25  4,6  1,9  662  26,5**  52,0** | 35  5,8  2,8**  667  19,1  52,5** |
