# Supplementary material for: Time-trends in the utilization of decentralized mental health services in Norway - A natural experiment: The VELO-project
Source: Int J Ment Health Syst. 2010 Mar 31;4:5. doi: 10.1186/1752-4458-4-5 (PMC2861015; doi:10.1186/1752-4458-4-5)
Supplement: Additional file 3 — Inpatient-population characteristics in a local-bed system versus a central-bed system. The years of 2003 and 2006. [file 1752-4458-4-5-S3.DOC]

**Additional file 3**

Inpatient-population characteristics, a local-bed system versus a central-bed system. The years of 2003 and 2006.

|  | | **Local-bed system** | | **Central-bed system** | |
| --- | --- | --- | --- | --- | --- |
|  |  | 2003 | 2006 | 2003 | 2006 |
| **Age** |  | 40.3 | 41.5 | 39.5 | 43.4 |
| **Gender** | Male   - N - 1/1000 inh.   Female   - N - 1/1000 inh. | 57  3.1**  82  4.5 | 58  3.2  99  5.4** | 62  4.9**  53  4.2 | 56  4.4  46  3.6** |
| **Diagnosis** | Substance-abuse   - N - 1/1000 inh. | 6  0.33** | 15  0.82* | 18  1.42** | 21  1.65* |
|  | Psychosis   - N - 1/1000 inh. | 45  2.45 | 53  2.91 | 25  1.97 | 30  2.36 |
|  | Affective disorders   - N - 1/1000 inh. | 31  1.69 | 36  1.98 | 28  2.20 | 23  1.81 |
|  | Anxiety   - N - 1/1000 inh. | 31  1.69 | 39  2.14** | 30  2.36 | 15  1.18** |
|  | Others   - N - 1/1000 inh. | 14  0.76 | 9  0.49 | 14  1.1 | 7  0.55 |
|  | Psych.examination   - N - 1/1000 inh. | 12  0.65 | 5  0.27 | -  - | 6  0.47 |
