# Supplementary material for: Time-trends in the utilization of decentralized mental health services in Norway - A natural experiment: The VELO-project
Source: Int J Ment Health Syst. 2010 Mar 31;4:5. doi: 10.1186/1752-4458-4-5 (PMC2861015; doi:10.1186/1752-4458-4-5)
Supplement: Additional file 4 — Predictors of inpatient treatment (no/yes) in a local-bed system. The years of 2003 and 2006. Logistic regression model. [file 1752-4458-4-5-S4.DOC]

**Additional file 4**

Predictors of inpatient treatment (no/yes) in a local-bed system. The years of 2003 and 2006. Logistic regression model.

| **Variable** | **2003**  N = 675 | | | **2006**  N = 742 | | |
| --- | --- | --- | --- | --- | --- | --- |
|  | **B** | **Sig.** | **Odds Ratio** | **B** | **Sig.** | **Odds Ratio** |
| **Patient characteristics**   - Gender (male = 1, female = 2), - Age | -.023  .019 | .919  .033 | .978  1.020 | .237  .009 | .282  .243 | 1.267  1.009 |
| **Diagnosis** (no = 0, yes =1)   - Substance abuse - Psychosis - Affective disorders - Anxiety - Psychiatric examination | .264  1.831  -.281  -.626  -.992 | .666  .000  .467  .096  .025 | 1.302  6.240  .755  .535  .371 | 1.131  2.364  -.016  -.213  -1.211 | .027  .000  .970  .619  .046 | 3.097  10.636  .984  .808  ..298 |
| **Treatment utilization**   - Outpatient consultations - Days in Day-hospital | .052  -.102 | .000  .738 | 1.053  .903 | .045  .021 | .000  .352 | 1.046  1.021 |
| Constant | -2.286 | .000 | .102 | -2.382 | .000 | .092 |
