# Supplementary material for: Time-trends in the utilization of decentralized mental health services in Norway - A natural experiment: The VELO-project
Source: Int J Ment Health Syst. 2010 Mar 31;4:5. doi: 10.1186/1752-4458-4-5 (PMC2861015; doi:10.1186/1752-4458-4-5)
Supplement: Additional file 5 — Predictors of inpatient treatment (no/yes) in a central-bed system. The years of 2003 and 2006. Logistic regression model. [file 1752-4458-4-5-S5.DOC]

**Additional file 5**

*Predictors of inpatient treatment (no/yes)* *in a central-bed system. The years of 2003 and 2006. Logistic regression model.*

| **Variable** | **2003**  N = 532 | | | **2006**  N = 607 | | |
| --- | --- | --- | --- | --- | --- | --- |
|  | **B** | **Sig.** | **Odds Ratio** | **B** | **Sig.** | **Odds Ratio** |
| **Patient characteristics**   - Gender (male = 1, female = 2) - Age | -.389  -.003 | .093  .734 | .678  .997 | -.315  .023 | .224  .014 | .729  1.024 |
| **Diagnosis** (no=0, yes=1)   - Substance abuse - Psychosis - Affective disorders - Anxiety - Psych. examination | 1.503  1.684  .823  .356  -1.944 | .001  .000  .025  .311  .998 | 4.497  5.385  2.277  1.428  .000 | 1.700  2.158  1.056  .017  -1.129 | .001  .000  .028  .973  .051 | 5.475  8.655  2.874  1.017  .323 |
| **Treatment given**   - Outpatient consultations - Days in Day-hospital | -.030  -.014 | .074  .269 | .970  .986 | -.051  .004 | .034  .823 | .950  1.004 |
| Constant | -1.390 | .004 | .249 | -2.708 | .000 | .067 |
